# Protocol of the Nutritional, Psychosocial, and Environmental Determinants of Neurodevelopment and Child Mental Health (COINCIDE) study

**DOI:** 10.12688/wellcomeopenres.22817.2

**Published:** 2024-11-22

**Authors:** Eunice Lobo, Deepa R., Siddhartha Mandal, Jyothi S. Menon, Aditi Roy, Shweta Dixit, Ruby Gupta, Sumathi Swaminathan, Prashanth Thankachan, Supriya Bhavnani, Gauri Divan, Poornima Prabhakaran, Onno CP van Schayck, Giridhara Rathnaiah Babu, Prashanth Nuggehalli Srinivas, Debarati Mukherjee, COINCIDE consortium

**Affiliations:** 1 Indian Institute of Public Health-Bengaluru, Public Health Foundation of India, Bangalore, India; 2 Institute of Public Health Bengaluru, Bengaluru, Karnataka, India; 3 Family Medicine, Care and Public Health Research Institute (CAPHRI), Maastricht University, Maastricht, Limburg, The Netherlands; 4 Centre for Chronic Disease Control, New Delhi, India; 5 Centre for Health Analytics Research and Trends (CHART), Trivedi School of Biosciences, Ashoka University, Sonipat, India; 6 Division of Nutrition, St. John's Research Institute, Bangalore, India; 7 Child Development Group, Sangath, New Delhi, India; 8 Department of Population Medicine, College of Medicine, QU Health, Qatar University, Doha, Qatar

**Keywords:** nutrition, early-life stress, air pollution, pesticide, cognitive development, child mental health, nurturing care framework, global South

## Abstract

**Background:**

Over 250 million children are developing sub-optimally due to their exposure to early life adversities. While previous studies have examined the *independent* effects of nutritional status, psychosocial adversities, and environmental pollutants on children’s outcomes, little is known about their interaction and cumulative effects.

**Objectives:**

This study aims to investigate the independent, interaction, and cumulative effects of nutritional, psychosocial, and environmental factors on children’s cognitive development and mental health in urban and rural India. It also seeks to explain pathways leading to inequities in child outcomes at the individual, household, and neighbourhood levels.

**Methods:**

A mixed-methods prospective cohort study will be conducted on 1600 caregiver-child dyads (child age 3–10 years) in urban and rural India. Nutritional status, psychosocial adversities, environmental pollutants, and child mental health outcomes will be assessed using parent-report questionnaires. Performance-based measures will be used to assess cognitive outcomes. Venous blood and urine samples will be used to measure nutritional and pesticide biomarkers in 500 children. Indoor air pollution will be monitored in 200 households twice, during two seasons. Multilevel regression, weighted quantile sum regression, and Bayesian kernel machine regression will assess the individual and combined effects of exposures on child outcomes. Thematic analysis of in-depth interviews and focus group discussions will explore pathways to middle-and late childhood development inequities.

**Discussion:**

The data will be used to formulate a Theory of Change (ToC) to explain the biological, psychosocial, and environmental origins of children’s cognitive and mental health outcomes across the first decade of life in diverse Indian settings, which can inform interventions targets for promoting children’s outcomes beyond the first 1000 days, potentially generalizable to similar under-resourced global settings. The COINCIDE research infrastructure will comprise a valuable global health resource, including prospective cohort data, validated study tools, and stored biological and environmental samples for future studies.

## Plain language summary

Globally, millions of children face developmental challenges while growing up in adverse environments, including poor nutrition, abusive or neglectful relationships, and high pollutant exposure. Although these individual factors impact children’s development, their combined effects on child outcomes, particularly in developing countries, is less understood. This study examines how nutrition, psychological stress, and exposure to air pollution, pesticides, and heavy metals, independently and collectively influence children’s cognitive abilities and mental health in urban and rural India. Additionally, it will examine pathways leading to developmental inequalities among different individuals, households, and communities.

This study will involve 1600 mother-child pairs when children are 3–9 years old, recruited from existing study cohorts in urban and rural India. Participants will answer comprehensive questions about their children’s nutrition, psychological stress, pollutant exposure, and mental health. Children's cognitive abilities will be assessed using performance-based measures, such as activity sheets and video games. Blood and urine samples will be collected from 500 children to measure nutrition and pollution markers. Indoor air pollution will be monitored twice in 200 households during summer and winter. Advanced statistical methods will be used to analyse and interpret the individual and combined effects of these various exposures on children's cognitive and mental health outcomes. Interviews and group discussions will provide deeper insights into why certain individuals, households, and communities bear a disproportionate burden of poor child development.

Our findings will inform a theoretical framework that explains how biological, social, and environmental factors affect children's outcomes through their first decade of life in diverse Indian settings, potentially applicable to similar global settings. This framework will then be used to identify intervention targets to improve children's outcomes. The COINCIDE project will also contribute valuable resources for global health research, including high-quality prospective data, study tools, and biological samples that could be leveraged in future studies.

## Introduction

While the Millennium Development Goals focused on reducing child and maternal mortality ^1^, the Sustainable Development Goals pivoted their focus to children thriving ^2^. Specifically, Target 4.2 calls for more investment in children’s health, development, learning, and psychosocial well-being, as over 250 million children worldwide are at risk of not achieving their developmental potential due to their exposure to a wide range of early life adversities. India has the highest number of children developing sub-optimally (64.3 million) ^3^. According to the Annual Status of Education Report (ASER) 2022 ^4^, only a fifth of the children enrolled in grade III in India can read grade II level texts, and only a quarter can perform subtraction. Furthermore, a recent global meta-analytic review of 708,561 individuals revealed that the peak age of onset for *any* mental health disorder is 14.5 years ^5^. While most mental health disorders have their onset during childhood or adolescence, few prospective studies have measured and tracked mental health outcomes from an early age. Therefore, the dearth of prospectively measured high-quality mental health data from early childhood onwards has grave implications for our ability to intervene early, and consequently, on the health and economic outcomes of the affected individuals throughout their lives. 

Childhood is a period of rapid growth and development of cognitive, language, motor, and social-emotional skills ^3, 6, 7^. It comprises a critical and sensitive period because environmental inputs play a significant role in shaping the rapidly developing brain ^6, 8^. Consequently, exposure to adversity in early life leads to a cascade of negative developmental consequences affecting cognitive, academic, and mental health outcomes during childhood, which then predicts poor health, quality of life, and human capital development during adulthood ^9– 12^. For example, a systematic review and meta-analysis concluded that cognitive development scores in the first two decades of life were inversely associated with mortality rates measured over the next five decades ^13^. Therefore, it is critical to measure and track child development outcomes and their determinants to inform policies and interventions geared towards reducing the global burden of physical and mental health disorders, and to meet the SDG targets.

Currently, there remains a significant gap in high-quality data from diverse contexts on factors affecting children’s development beyond the first three years of life, especially in the Global South ^14, 15^. Consequently, there is little evidence on specific intervention targets that can promote children’s outcomes during middle and late childhood, even though key indicators measured during this period (e.g., cognitive development) are more reliable in predicting later health, academic, and economic outcomes ^16^. Furthermore, emerging insights into the early onset of mental health disorders call for increased investments in long-term prospective studies that examine early life factors that influence later mental health outcomes ^17^. 

Suboptimal neurodevelopment and adverse child mental health outcomes are important public health challenges in India that directly jeopardize benefits that can be accrued from its significant demographic dividend ^18^. Previous research has shown that nutritional status ^19, 20^, psychosocial adversities ^21, 22^, and environmental pollutants ^23– 25^ independently impact cognitive development and mental health in children, and some evidence suggests that these factors can moderate the effects of others. For example, while environmental pollutants such as heavy metals and pesticides have been associated with neurodevelopmental deficits and behavioural problems in children ^26^, their effects may be modified by nutritional status ^27– 29^ and/or psychosocial environment ^30, 31^. Therefore, to generate a comprehensive and nuanced understanding of the complex nature of children’s exposures, and their combined and intersectional effects on child outcomes, a team science approach is essential because it requires the combined resources and skills cross-cutting multiple disciplines. The Nutritional, Psychosocial, and Environmental Determinants of Neurodevelopment and Child Mental Health (COINCIDE) study was initiated in October 2021, with a team of experts in nutrition, environmental health, social science, child development, and advanced statistics to address these gaps.

### Specific aim and hypothesis

In collaboration with this multidisciplinary and multi-institutional team of researchers based in India, the COINCIDE study aims to: (1) evaluate the independent, interaction, and cumulative effects of nutritional, psychosocial, and environmental determinants of cognitive development and mental health in 3–10-year-old children in rural-North and urban-South India; and (2) explore pathways that result in child development inequities at the individual, household, and neighbourhood levels through an in-depth qualitative inquiry.

Using this unique and comprehensive database encompassing the first decade of life of urban and rural Indian children, the COINCIDE study will develop an evidence-based and contextualized Theory of Change (ToC) that explains the pathways through which key determinants of children’s cognitive development and mental health outcomes interact with axes of health inequities (socioeconomic status and gender), leading to differential child outcomes in diverse Indian settings. The ToC framework can help identify interventions for the development and refinement of evidence-based and contextualized interventions that are potentially generalizable to similar settings in the Global South. Furthermore, the research infrastructure of the COINCIDE study will serve as a valuable global health resource by generating quantitative and qualitative data to examine the determinants of children’s cognitive development and mental health outcomes from two diverse birth cohorts in India, as well as stored biological samples suitable for genetic, epigenetic, biochemical, and metabolomic assessments in future studies. These efforts will contribute to advancing the agenda of optimizing the developmental potential of the most disadvantaged children residing in low-resource settings. The benefits accrued during the foundational years will help improve health, academic, and economic outcomes throughout life.

### Hypothesis

We hypothesize that

1.   Specific adversities experienced within the nutritional, psychosocial, and environmental domains will have differential impacts on children’s cognitive and/or mental health outcomes.

2.   Each type of adversity will have a ‘sensitive window of exposure’ wherein exposure to this factor in this critical time window will have long-lasting effects on child outcomes during middle- and late-childhood.

3.   Increasing number of adversities will be associated with poor cognitive and mental health outcomes across all age groups in both urban and rural settings. However, the nature of adversities will differ across the urban and rural settings.

4.   The psychosocial environment of the child will moderate the effects of adverse nutritional and environmental pollutant exposures during middle- and late-childhood. Specifically, a nurturing and responsive environment will protect against the harmful effects of adverse nutritional or environmental pollutant exposures, while an abusive or neglectful environment will exacerbate their negative effects.

In addition to the hypothesis-driven research described above, we will also attempt to identify the combination of the most important predictors of child outcomes in different age groups and settings using a data-driven machine learning approach. The outcome will be tested in a naïve test sample that does not contribute to training the machine learning models.

## Methods

### Study design and setting

**Figure 1.**
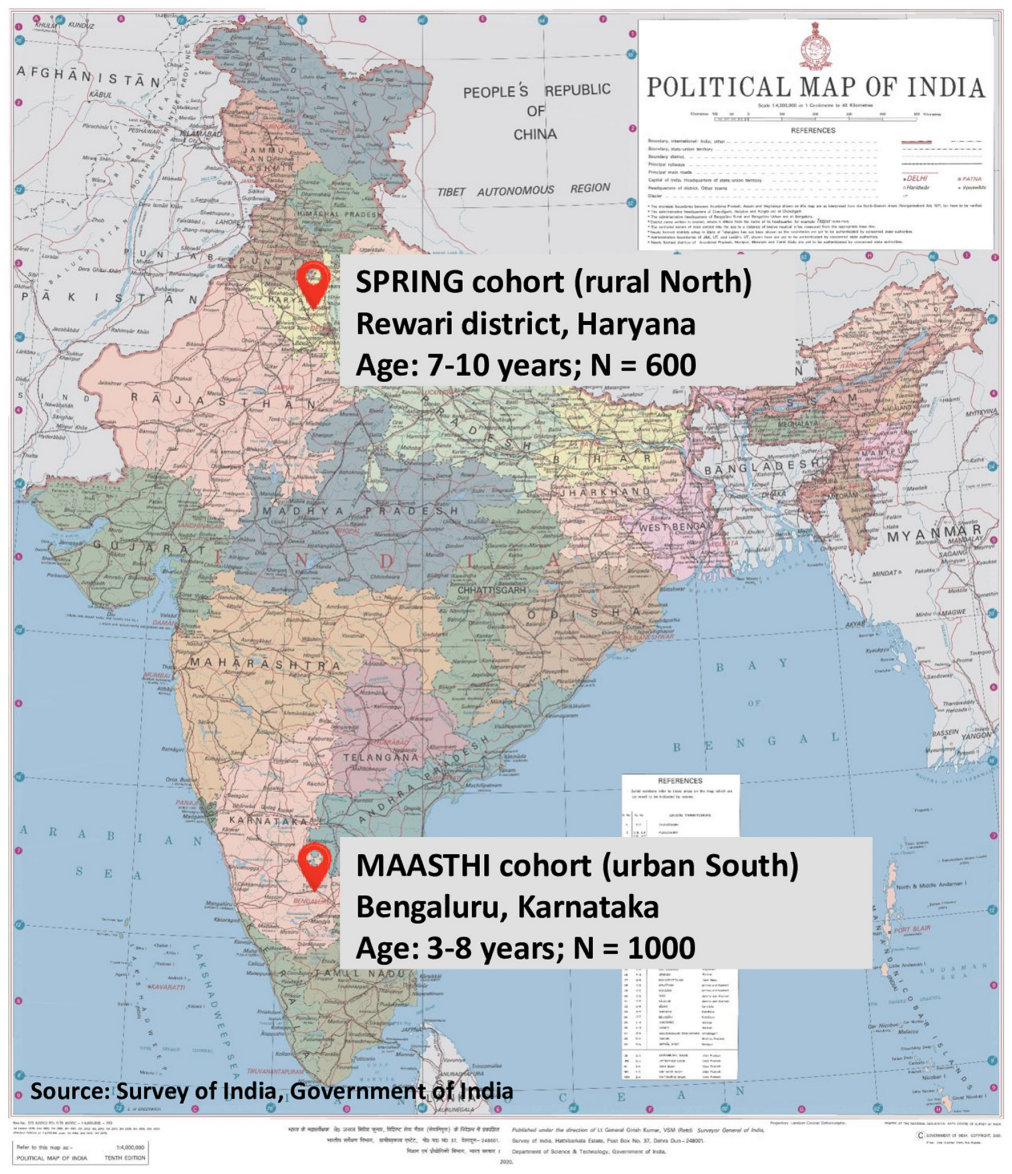
Location, sample size, and child age range of the SPRING and MAASTHI birth cohorts.

The COINCIDE study is nested within two existing birth cohorts in urban South (Bengaluru, Karnataka) and rural North (Rewari, Haryana) India ( Figure 1). Both birth cohorts have been described elsewhere ^32– 34^ and are briefly described below. The current study is a prospective cohort study that will extend the follow-up of caregiver-child dyads when children are 3–10-years old.

*MAASTHI birth cohort in Bengaluru, Karnataka (urban South India):* The Maternal Antecedents of Adiposity and Studying the Transgenerational Role of Hyperglycaemia and Insulin (MAASTHI) birth cohort was set up in 2016–2019 in Bengaluru, Karnataka to investigate the relationship between maternal glucose levels, nutritional status, and psychosocial exposures, on infant adiposity and development outcomes ^32^. Research from MAASTHI has demonstrated the intergenerational impact of maternal factors on birth and infant outcomes, including demonstrations that gestational obesity, maternal nutritional status, gestational diabetes mellitus, breastfeeding practices, and maternal psychosocial stress, adversely impact neonatal adiposity, birth weight, anthropometry, and child development outcomes during infancy ^35– 49^.

*SPRING birth cohort in Rewari, Haryana (rural North India):* The Sustainable Programme Incorporating Nutrition and Games (SPRING) birth cohort was set up in 2014 in Rewari, Haryana, to test a parenting intervention with messages on nutrition and cognitive stimulation to promote child growth and development outcomes in the first 1000 days of life ^50^. It incorporated an early life stress sub-study (SPRING-ELS) ^51^ that assessed the impact of 22 psychosocial adversities in the perinatal period on child outcomes. SPRING demonstrated inverse associations between psychosocial adversities and children’s growth and development outcomes at 18- and 36-months of age. Cumulative early-life adversity scores were positively associated with children’s hair cortisol levels, thereby indicating a putative mechanistic link between adversities and neurodevelopment through the hypothalamus-pituitary-adrenal axis ^52^.

According to the fifth round of the National Family Health Survey (NFHS-5) in Bengaluru and Rewari districts ^53, 54^, while 97.2% of households used clean cooking fuel in Bengaluru, only 54.9% did so in Rewari. Furthermore, twice the number of women aged 15–19 years in Rewari district were already mothers or pregnant at the time of the survey compared to those in Bangalore, and pregnant women in Rewari were less likely to attend all four antenatal visits as mandated by the Government of India. Therefore, the MAASTHI and SPRING cohorts represent diversity in location (urban/rural) and cultural context (north/south India), which may lead to potential differences in their diet and food security, opportunities for learning, cooking fuels used, and exposure to pesticides ^55– 57^.

### Study sample

The study sample covers the Pregnancy-10-years-age range across both cohorts.

***Historical data***

Historical data since pregnancy on several exposure and outcome measures relevant to the COINCIDE study are available from both cohorts ( Table 1).

**Table 1.** Historical data available in the MAASTHI and SPRING cohorts.

MAASTHI birth cohort, urban South India	SPRING birth cohort, rural North India
• Sociodemographic details, 24-hour dietary recall, dietary habits, use of tobacco and alcohol (participant and spouse), and physical activity in the women. • Family history of diabetes and other cardiovascular diseases, obstetric history • Oral Glucose Tolerance Test • Maternal and child anthropometry • Haemoglobin status, depressive symptoms using the Edinburgh Postnatal Depression Scale (EPDS), and social support in all women • Particulate matter 2.5 (PM _2.5_), PM _10_, and carbon monoxide exposure was measured in a sub-set of mothers during pregnancy using personal air pollution monitors • Follow-up visits involved assessing anthropometry and the health profile of mothers and children. • Plasma & serum samples from pregnant women (2 ^nd^ trimester) have been stored for future research.	• Sociodemographic details, including geotagging of households for their locations, dietary habits of mother and child, and paternal use of tobacco and alcohol. • At-home ‘learning and playing opportunities’ for children, screening for depression using Patient Health Questionnaire 9 (PHQ-9) and social support among the mothers, and for mothers who faced gender-based violence. • Child anthropometry, adversities, hair, and saliva cortisol levels. • Electroencephalogram (EEG) data among parents (mothers and fathers) and children (at the age of 36 and 60 months). • Developmental assessment on an E-Platform (DEEP) during the children’s follow-up at 3 & 5 years. • Follow-up visits involved assessing anthropometry and the health profile of mothers and children aged seven years and nine years. • A sub-study on early life stress assessed the impact of 22 psychosocial adversities in the perinatal period on child outcomes.

The MAASTHI cohort includes 2962 pregnant women and their children, recruited from public health facilities in Bengaluru. Between 2016–2019, participants were followed up during pregnancy, at 14-weeks, and annually until the oldest children were four years old. The number of children followed up at one year was 2371, while 2463, 2245, and 1500 were followed up as they reached two, three, and four years of age respectively. In the MAASTHI cohort, women moved to their maternal home for care and support during pregnancy and stayed there for a few months after delivery, in some cases, for as long as a year, especially if they were giving birth for the first time. Since annual follow-ups were conducted irrespective of the completion of previous follow-ups, more mother-child dyads were followed up when children were two years old, compared to when they were one.

Venous blood was collected from the mothers during pregnancy. A total of 32,615 plasma and serum aliquots were stored for future analysis ^32^. Data on particulate matter 2.5 (PM _2.5_), PM _10_, and carbon monoxide exposure were measured in 297 mothers during pregnancy using personal air pollution monitors ^48^.

The SPRING cohort enrolled 1726 participants from 120 villages in the Rewari district of Haryana, India in 2014–2015. Primary outcome assessments (anthropometric measures and developmental assessments using Bayley’s Scales of Infant and Toddler Development, 3rd Edition ^58^) were completed in 1443 children at 18-months of age. Of these 1443 children, 1359 were followed up at 3-years and 1243 at 5-years of age. 

### Sample size, inclusion, and exclusion criteria for the COINCIDE study

The sample for the COINCIDE study includes a subset of 1000 mother-child dyads (child age 3–8 years) from the MAASTHI cohort and 600 mother-child dyads (child age 7–10 years) from the SPRING cohort (total N = 1600) ( Figure 1). The sub-sample is recruited such that they are representative of the original cohorts with respect to geographical distribution, child sex, and household socioeconomic status. Children with congenital anomalies, severe developmental disorders, or any other condition that impairs their ability to meaningfully engage with tablet-based assessments (such as severe vision, hearing, or motor disorders) were excluded since one of the outcome assessments involve the use of a gamified tablet-based assessment ^59, 60^. (See *quantitative data collection* sub-section for details). Details regarding the sample size and power calculations are provided in Table 2.

**Table 2.** Sample size and power calculations for the COINCIDE study. Required sample size per group, for detecting a difference of delta (d) per unit standard deviation (SD) in time averaged response, between two exposure groups, with 90% power and 5% level of significance. We show sample size computations for three different values of within individual correlation (rho) under an exchangeable correlation structure.

Domains of exposure	Specific exposure	Measurement	Outcome	SD-outcome	Difference in units (d)	Delta (d/SD)	rho = 0.2	rho = 0.4	rho = 0.6	Reference
Nutritional	Dietary available Iron (Fe)	24-hour food recall	RCPM	0.598	6.6×10−2	0.11	1034	1207	1379	64
	Body Iron (mg/kg)	Venous blood	RCPM	4.67	-0.75 (-1.44; -0.06)	-0.16	488	570	651	65
Psychosocial	Adverse childhood experience	Questionnaire	RCPM	10.5	6.5	0.62	33	38	44	66

RCPM: Raven’s coloured progressive matrices; d: difference in units refers to the difference in Standard Deviations (SD)

### Study timeline ( Figure 2)

**Figure 2.**
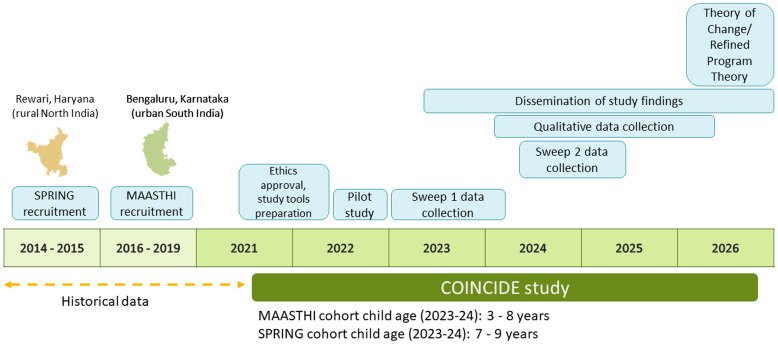
Summary of COINCIDE field activities and objectives.

Quantitative data will be collected at two time points (hereafter referred to as Sweep 1 and 2). The first sweep of quantitative data collection for this study commenced in January 2023 in the SPRING cohort and April 2023 in the MAASTHI cohort after obtaining ethical approval from the institutional ethics committees. Data collection for Sweep 1 was completed in April 2024. Sweep 2 data collection is scheduled to begin in September 2024 and completed by August 2025. Qualitative data collection commenced in February 2024 and will continue until 2026. Data management, analysis, and reporting will continue until the end of the project period (September 2026). 

### Data collection

Consent for new data collection was obtained by leveraging previous long-standing relationships with the cohort participants. Field assessors read the participant information sheet during the consent process. A recruitment flyer with visual descriptions of key study procedures was used to support participants in comprehending study procedures and the associated risks and benefits. All documents to re-enrol MAASTHI and SPRING participants in the COINCIDE study have been approved by the institutional ethics committees of participating institutes.

***Quantitative data collection***

The list of exposures, outcomes, and confounder measures, along with the validated questionnaires and tools used to measure them, are listed in Table 3, and briefly described below. During data collection, questionnaires are administered to the primary caregiver of the child (mothers in most cases). Paper and tablet-based cognitive assessments are administered directly to the child and the mother (details below).

**Table 3.** Exposure and outcome assessment domains and tools for data collection.

Variable	Respondent	Approach	Tool
***Socio-demographic or health***			
Registration form	Mother/Caregiver	Interview	Questionnaire
Demographics	Mother/Caregiver	Interview	Adapted from the Demographic and Health Survey questionnaire ^70^
Socio-economic status	Mother/Caregiver	Interview	Questionnaire
Maternal health and morbidity	Mother/Caregiver	Interview	Questionnaire
Paternal health and morbidity	Mother/Caregiver	Interview	Questionnaire
Child health and morbidity	Mother/Caregiver	Interview	Questionnaire
***Nutrition status***			
Anthropometry	Child	Direct measurement	As per standardized protocol ^71^
24-hr food recall (child)	Mother/Caregiver	Interview	Standardized Questionnaire ^72^
Household-level dietary diversity	Mother/Caregiver	Interview	Household level dietary diversity ^73^
Household-level food insecurity	Mother/Caregiver	Interview	Food insecurity experience scale ^74^
Blood Biomarkers	Child	Blood sample collection and analysis	As per standardized protocol
Body fat and fat-free mass (250 children in the MAASTHI cohort)	Child	Air Displacement Plethysmography	BOD POD test
***Psychosocial environment***			
Maternal mental health (SPRING cohort)	Mother/Caregiver	Interview	Patient Health Questionnaire-9 (PHQ-9) ^75^
Maternal mental health (MAASTHI cohort)	Mother/Caregiver	Interview	Edinburgh Postnatal Depression Scale (EPDS) ^76^
Maternal social support	Mother/Caregiver	Interview	Validated questionnaire ^77^
Maternal adversity	Mother/Caregiver	Interview	Adapted from the SPRING Early Life Stress Questionnaire ^36^
Maternal IQ	Mother/Caregiver	Performance	Raven’s Standard Progressive Matrices ^69^
Parent-child relationship	Mother/Caregiver	Interview	Child-Parent Relationship Scale (CPRS) ^78^
Opportunities for learning and play	Mother/Caregiver	Interview	Family Care Indicators (FCI) ^79^
Psychosocial adversities in the child	Mother/Caregiver	Interview	Child Psychosocial Adversity Scale (CPAS) ^80^
Gender norms attitude	Mother/Caregiver	Interview	Questionnaire used in SHINE cohort (Zimbabwe) ^68^
Child education and screen exposure	Mother/Caregiver	Interview	Questionnaire
***Environmental Health***			
Indoor Air Pollution assessment	Mother/Caregiver	Interview	Questionnaire developed by Ashoka University ^81^
		Direct measurement (monitoring data)	Indoor air pollution monitoring as per standardized protocol
Ambient Air Pollution assessment		Modelling	Machine learning based ensemble modelling
Heavy metals/pesticide exposure questionnaire	Mother/Caregiver	Interview	Questionnaire developed by Ashoka University
Pesticide/Heavy metal	Child	Urine sample collection and analysis	As per standardized protocol
***Outcome assessments***			
Cognitive abilities	Child	Performance	Developmental Assessment on an E-Platform (DEEP) ^59, 60, 82^
		Performance	Raven's Coloured Progressive Matrices ^69^
Child mental health	Mother/Caregiver	Interview	Strengths and Difficulties Questionnaire (SDQ) ^83^
Literacy and numeracy	Child	Performance	Annual Status of Education Report (ASER) ^84^
Developmental milestones/screening	Mother/Caregiver	Interview	Rastriya Bal Swasthya Karyakram (RBSK) questionnaire ^85^

***Questionnaires***

Most of the study questionnaires and measures listed in Table 3 have either been used in the cohorts during earlier rounds of data collection, or have been used by the COINCIDE expert groups in Indian study settings. Some were selected from the literature demonstrating prior use in Indian settings ^61– 63^. Children’s mental health, one of the primary outcome measures in COINCIDE, is measured using the Strengths and Difficulties Questionnaire (SDQ) in children aged ≥4 years. The SDQ consists of 25 items that measure five domains: emotional problems, conduct problems, hyperactivity, peer problems, and prosocial behaviour. The scores for the first four domains are summed to derive the ‘Total Difficulties Score’. Based on previous work in India ^67^, boys scoring >22 and girls scoring >23 will be referred for further assessments and counselling. The SHINE cohort study demonstrated significant associations between gender norm attitude and maternal caregiving behaviours in a rural Zimbabwean (Global South) setting ^68^. The questionnaire to assess maternal gender norms attitude was obtained from a collaborator in the SHINE study and will be used for the first time in India. 

All questionnaires and study tools were independently translated into the local languages ( *Hindi* for the SPRING cohort and *Hindi* and *Kannada* for the MAASTHI cohort) by two translators (T1 and T2). Both translators reviewed both the versions and agreed on the best version (T1/2). A third translator, blind to the original English version of the tool, back-translated the T1/2 version into English. Senior research team members at both the cohort sites compared the original English version of the tool with the back-translated version to ensure that the meaning of each item was retained and accurately reflected the construct the original tools intended to capture.

### Brief description of direct child and maternal measures

***Maternal Intelligent Quotient (IQ)***

Raw scores of the Raven’s Standard Progressive Matrices (RSPM) will be used to measure maternal IQ ^69^. RSPM is a non-verbal ability test comprising five sets of 12 items each, with progressively increasing difficulty levels.

***Child cognitive development, literacy, and numeracy measures***

Cognitive development of children aged 3–7 years is measured using a tablet-based gamified assessment tool named DEvelopmental Assessment on an E-Platform (DEEP) ^59, 60, 82^. DEEP has fourteen games, each with multiple levels of difficulty, woven together through a first-person narrative. The DEEP tool measures multiple cognitive skills, including manual processing speed, manual coordination, hand-eye coordination, attention, response inhibition, reasoning, visual form perception, visual integration, and memory. Children aged ≥4 years are also be tested using the Raven’s Coloured Progressive Matrices (RCPM) ^86, 87^. This tool measures fluid intelligence and non-verbal reasoning abilities of 4–11-year-old children, and comprises three sets of 12 items each of increasing difficulty. Norms are available for the Indian population ^88^.

Literacy and numeracy is measured using the pre-school version of the Annual Status of Education Report (ASER) tool that has previously been used in India ^84^. Stimuli is presented using flipbooks. Items are comparable to widely used literacy-numeracy measurement tools such as the Early Grade Reading Assessment (EGRA) ^63^. Continuous scores on DEEP, RCPM, and ASER tools will be used as three independent measures of cognitive development.

***Child anthropometry***

Height, weight, head circumference, and mid-upper arm circumference (MUAC) are measured using standard techniques recommended by the World Health Organization ^89^. Height is measured using the SECA 213 portable stadiometer. Weight is measured using the SECA 803 weighing scale. Head circumference and mid-upper arm circumference is measured using the SECA 201 measurement tape. Field assessors in MAASTHI were trained and certified by the nutrition expert group in COINCIDE to collect anthropometry data. The SPRING cohort followed a train-the-trainer model, with the anthropometry expert carefully monitoring the training and certification processes. The specific exposure measures relevant to COINCIDE will be height-for-age-z-scores, weight-for-age-z-scores, and Body Mass Index-for-age-z-scores (BMI-for-age), which will be computed using the World Health Organization's child growth standards ^90^. Underweight, stunting, and wasting will be defined as two standard deviations below the median of the WHO standard curves.

***Biological samples from children***

Ten millilitres (ml) of venous blood (6 ml in plain red cap vacutainer, 4 ml in purple cap EDTA vacutainer) and at least 20 ml of urine samples was collected from 500 children during the first sweep of data collection (250 children at each site). In the MAASTHI cohort, biological samples were collected by trained phlebotomists from St. John’s Research Institute (study partner) through camps organized in public health facilities close to participants’ residences. In the SPRING cohort, biological samples were collected by a trained phlebotomist through household visits.

Blood and urine samples were transported to laboratories using cold-chain protocols for processing and storage within six hours of collection. Vials containing blood samples were covered with aluminium foil to prevent breakdown of light-sensitive biomarkers. The vials were placed in an ice box lined with gel packs, ensuring that they were not in direct contact with the gel packs. Blood samples collected in the red-capped plain tubes were centrifuged at 4°C at 3500 rpm for 10 minutes in a cold centrifuge. The separated serum samples were then aliquoted into five black cryovials of 500 microliters each, and the clot sample was stored as a separate aliquot.

The sample in the EDTA tube was first used to analyse the complete blood profile, including haemoglobin levels. 500 microliters of whole blood were aliquoted and stored for heavy metals assessment. The remaining sample was centrifuged in a cold centrifuge at 4°C for 3500 rpm for 10 minutes, and then aliquoted and stored at -80°C (one aliquot of buffy coat, three aliquots of plasma, and one aliquot of packed cell).

Stored plasma and serum samples will be used to measure biomarkers of micronutrients and heavy metals (see list in Table 4). This data will be used to determine multiple micronutrient deficiencies, iron deficiency, and inflammation levels in children. Whole blood samples will be analysed for heavy metal exposure using Inductively coupled mass plasma spectrometry (ICP-MS) ^91^.

**Table 4.** List of nutritional and pesticide biomarkers in the COINCIDE study.

Biological sample	Biomarkers
Blood (child) N=500 (250 at each site)	Ferritin
	Soluble, transferrin receptor (sTfR)
	C-Reactive Protein (CRP)
	Alpha(1)-acid glycoprotein (AGP)
	Vitamin A
	Vitamin B6
	Vitamin B12
	Folate
	Vitamin D
	T3
	T4
	Toxic metals such as Lead, Cadmium, etc.
Urine (child) N=500 (250 at each site)	Six Di-alkyl Phosphate (DAP) metabolites as a biomarker for organophosphate pesticide exposure
	Creatinine

At least 20 ml of urine samples were collected, placed in an insulated box lined with refrigerated gel packs, and shifted to the lab within six hours of collection. Three millilitres of urine sample were aliquoted into three tubes within eight hours of collection and stored at -80 ^o^C. Six common dialkyl phosphate (DAP) metabolites will be measured as biomarkers of organophosphate (OP) insecticides, a class of pesticides widely used in India in both agricultural and residential settings, using a previously validated method ( Table 4) ^92, 93^. Urinary creatinine concentration will be measured to adjust for dilution due to variation in urine volume, and will be adjusted in statistical models with pesticide exposure and child outcomes ^94^.

***Indoor air pollution***

Indoor PM _2.5_ concentration was assessed for 24 hours in 200 households (100 households at each site) using portable air pollution monitors (Personal DataRAM™ pDR-1500 Thermo Scientific, Inc.) ^95^. Monitoring was repeated in the same household across two seasons to account for seasonal variations ^81, 96^. Field assessors administered a post-monitoring questionnaire to record household activities during the monitoring period, and prepared a rough sketch of the household with the position of the monitor and the child’s bed marked on the sketch. The monitor was placed in a location where the child spent most of their time inside the household (typically this was the child’s bedroom). The monitor also collects gravimetric measurements, a subset of which will be subjected to gravimetric assessment to estimate particulate matter concentrations. This will be used to develop site- and season-specific calibration factors (or best-fit curve) and applied to the raw data to adjust the real-time measurements obtained from the instrument ^95, 97^.

***Qualitative data collection***

We will follow the principles of theory-driven inquiry ^98, 99^ to address the second objective: to explore pathways at the individual, household, and neighbourhood levels that result in inequities in child development outcomes. Two scoping reviews ^100^ will be conducted to comprehensively understand the role of caregiving practices and parental involvement in shaping children’s development outcomes. This information will be used to develop an initial program theory (IPT) and tools for the qualitative study specific to each site. The initial program theory (IPT) will be refined using emerging qualitative and quantitative evidence from each site to formulate an empirically validated refined program theory (RPT). The RPT will aim to explain plausible pathways through which risk factors of children’s development and mental health play out differentially in different social and economic contexts at the individual, household and neighbourhood levels ^101^.

Qualitative data will be collected using in-depth interviews (IDIs), focus group discussions (FGDs), key informant interviews (KIIs), stakeholder workshops, and field notes. Informed consent will be obtained from a sub-set of purposively selected participants for the in-depth interviews. We will ensure privacy and confidentiality during the interviews. The refined programme theory will be used to construct a Theory of Change (ToC) model through consultations and workshops with child development experts and other stakeholders. The ToC could guide the design of future interventions..

### Data management

The Research Electronic Data Capture (REDCap) application deployed on Android tablets ^102, 103^ is being used to collect quantitative data at both the cohort sites. The DEEP cognitive assessment tool is installed on the same Android tablet. Paper forms (consent forms, RCPM, RSPM, household sketches, etc.) are digitized and stored in a password-protected repository accessible only to senior researcher staff along with data downloaded from the REDCap mobile application. All audio-recorded interviews are securely stored in a password-protected repository with limited access only to senior research team members. In cases where consent is refused for audio recording, extensive field notes are taken and used for analysis.

### Assessor training and quality assurance

Data collection in both the sites is led by assessors trained by expert groups in COINCIDE. Training sessions included a combination of didactic lectures, role-play, and practice sessions. Assessors practiced administering the tools on non-cohort participants to gain proficiency before initiating data collection for the pilot study. The results from the pilot study were used to further refine standard operating procedures (SOPs), translations, the order in which tools are administered, identify and implement solutions to unexpected field-level challenges, and assessing the time burden of individual tools. SOPs were iteratively updated through the pilot study to address any threats to the accuracy of the data collected (e.g., translated version of an item was not clear to the participant), or challenges in administration (e.g., response options were difficult to explain to participants, branching logic errors in the REDCap data collection application). During data collection for the main study, the SOPs and REDCap forms refined and finalized based on learnings from the pilot study were used by the senior research members to conduct refresher training of field assessors every six months to ensure fidelity to the study protocol. At least 10% of all assessments were supervised during the first sweep of data collection by senior research team members to ensure consistency, and to provide ongoing performance feedback to the assessors. The same plan will be followed for Sweep 2. Senior team members travelled to both cohort sites to ensure consistency in data collection across sites. Inter-rater reliability for all assessments was assessed to ensure consistency in evaluations across field assessors.

The data management team conducts weekly reviews of the data collected by the field assessors or at the end of the workday to ensure that all digital records captured through REDCap and DEEP are uploaded to secure servers. Paper forms are scanned and archived in dedicated folders. The data management team also conduct verbal quality checks with the field assessors daily to record any deviations from the study protocol and note any new challenges faced. They also scrutinize the uploaded data and make a note of inconsistencies or errors on a weekly basis.

Weekly team meetings are held within and between the cohort sites to track data collection progress, identify inconsistencies in data, address any technical or administrative challenges experienced by the field teams, and resolve any data errors noted. The entire COINCIDE team, including the principal investigators, senior research team, and junior research assistants meet on a monthly basis to discuss study progress and mitigation strategies of existing challenges, plan the agenda for the next phase, and review drafts of ongoing manuscripts and conference abstracts or presentations.

### Analysis plan

***Data cleaning and pre-processing***

Data on all exposure and outcome measures will be cleaned, processed, and summarized as mean ± standard deviation (symmetric data), or median ± interquartile ranges (skewed data), or percentages (categorical variables), to examine between and within individual variability across the two sweeps of data collection. Individual cohort data will be analysed separately, followed by an examination of the similarities and differences between exposures, outcomes, and the relationship between them in the two cohorts. A dedicated data management team including senior statistical experts, study coordinators, site-specific coordinators, and supervisors conduct weekly meetings to collaboratively work on the development and management of the REDCAP database, address field-level data challenges (data entry, uploads to central servers, daily downloads for data backup and storage, etc.), and develop code for automated data cleaning, processing (generating summary scores, identifying missing data, etc.), and visualizations.

*Objective 1a: Independent effects of nutritional status, psychosocial adversities, and environmental pollutant exposures on cognitive development and mental health in 3–10-year-old children*

The effect of individual exposures modelled using linear or nonlinear (based on quintiles) formulations will be analysed using linear or generalized linear longitudinal regression models, with the main effects of the exposure (time-varying or invariant) on outcomes being assessed while accounting for relevant confounders. We will use mixed-effects models to incorporate individual-level differences in slopes and intercepts and the clustering of exposures by location as required. We will interpret the changes in the outcome scores (RCPM and DEEP for cognitive development and SDQ total and domain scores for child mental health) per unit standard deviation/interquartile range change in exposure (or between categories of exposures as relevant).

*Objective 1b: Combined effects of nutritional status, psychosocial adversities, and environmental pollutant exposures on cognitive development and mental health in 3–10-year-old children*

To assess the joint effects of two exposures (for example, maternal depression and ambient PM2.5 exposures), we will analyse multiplicative interactions between the two exposures within the longitudinal model framework. For more than two continuous exposures (for example, dietary micronutrients), we will leverage a recently developed methodology, Bayesian Kernel Machine Regression (BKMR), that models the complex, potentially nonlinear and non-additive exposure-response function between exposures from multiple domains and outcomes using flexible, functional forms approximated by kernel basis functions. These kernels attempt to shrink the estimated health effects in individuals with similar exposure profiles towards each other, thereby providing the joint effects of multiple exposures. For example, to determine the interactions of maternal depression with different indicators within exposure domains (e.g. ambient and indoor levels of PM _2.5_ and PM _10_ and nutritional biomarkers), we will utilize a combination of the above methodologies, specifically models of interaction, to arrive at quantitative effect estimates.

***Secondary analysis***

*Sensitive windows of exposure*

We will also assess the relative impact of exposures on outcomes across pre-specified windows of exposure (pregnancy, early-, mid-, and late-childhood periods defined as child age 0–3, >3–6, and >6–10 years respectively). Time-averaged exposure measures over these defined periods will be used to compare the effects of the same exposure across the first decade of life on cognitive development and mental health during late childhood, using generalized additive models with penalized splines of exposures. These models would explain potential non-linearities in the association between exposures and outcomes. Further, the strength of association between the exposures and outcomes across exposure windows would be analysed by incorporating distributed lags of exposure ^104^, which will be indicative of the importance of particular exposures in different developmental periods. Since average exposures at different periods would be correlated and fitting separate models for each time period may fail to account for other time periods, we will implement distributed lag models using time-varying exposure averages as predictors and outcomes as the dependent variable. These models account for all the periods simultaneously and assume that the association between exposure at a particular period and the outcome, controlling for the exposure at all other time periods, varies smoothly over time.

*Machine learning-based prediction of child outcomes*

We will develop a machine learning-based model to predict children’s cognitive, academic achievement, and mental health outcomes (scores based on direct child assessments and questionnaire-based tools) against predictor variables from the psychosocial, nutritional, and environmental domains. We will split the dataset for each study setting into training (70%) and validation (30%) datasets. Tree-based models are useful to model non-linearities and interactions between variables and would be our primary choice of algorithms. Random forests and gradient boosting models will be developed with internal cross-validation to tune the hyperparameters. Predictions from these algorithms on the validation dataset will be evaluated using measures of prediction accuracy such as R2 and root mean squared errors (RMSE).

### Scientific advisory group

A scientific advisory group comprising national and international experts on nutrition, psychosocial adversities, environmental health, and child development has been formalized to advise the COINCIDE team. They provide scientific oversight on research progress and outputs of the COINCIDE study team, while also providing mentorship on dissemination of results to a diverse range of stakeholders and planning short- and long-term goals for the COINCIDE team. The COINCIDE team communicates with the advisory group through monthly newsletters and dedicated annual meetings, and plan one-on-one meetings with one or a few advisors on a need basis. The Chair of the advisory group also provides independent feedback to the funder about the progress and impact of the study.

### Referral pathways for child and mother

**Figure 3.**
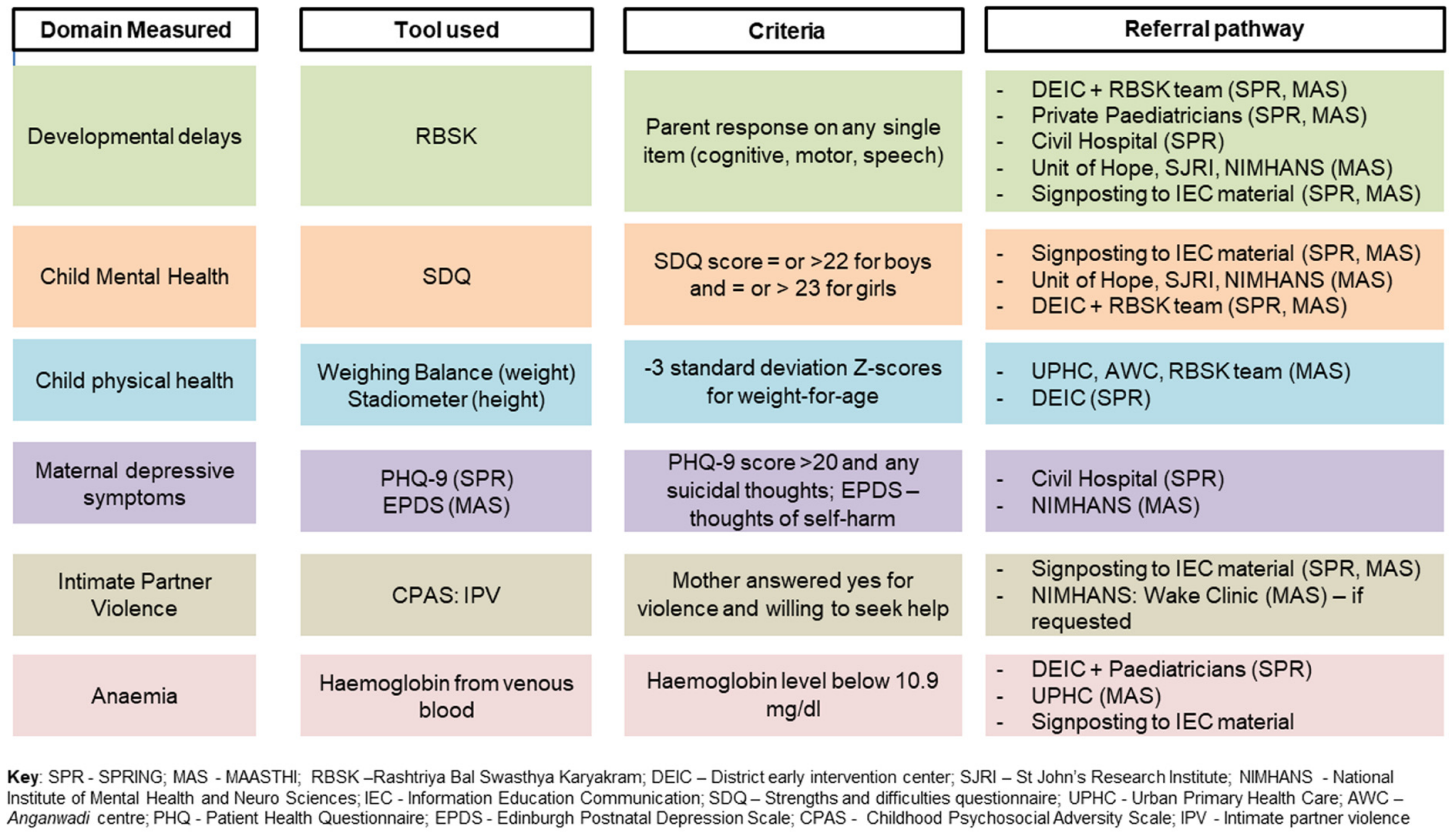
Criteria for referral and referral pathways in the COINCIDE study.

Both cohort sites have identified referral pathways where children and/or mothers are referred if they meet predefined referral criteria. For the MAASTHI cohort, this includes the National Institute of Mental Health and Neurosciences (NIMHANS), St. Johns’ Research Institute (SJRI), the District Early Intervention Centre (DEIC) of the Rashtriya Bal Swastha Karyakram (RBSK), and urban public health centres close to participant households. In Rewari, the Rewari Civil (District) Hospital, government health centres in the study area, and private paediatricians are part of the referral network. Figure 3 summarizes the criteria for the referrals across various domains.

### Developmental milestones/screening

Although not an outcome of our study, we included a questionnaire to screen for developmental delays and disabilities to identify children who would benefit from a referral for further assessment, diagnosis, and treatment as required. We used the *Rashtriya Bal Swasthya Karyakram* (RBSK) questionnaire for this purpose. This is a comprehensive screening tool aimed at early detection of the 4 'D's: defects at birth, diseases, deficiencies, and developmental delays, including disabilities, in children aged 0-18 years, to facilitate timely intervention and treatment.

The RBSK checklist is a structured tool used to screen children, and includes a series of questions designed to assess various domains of child health:

- Vision and auditory: Difficulty in seeing at day/ night; difficulty in hearing (without hearing aid)
- Motor development: Delays in walking, stiffness or weakness in limbs.
- Neurological concerns: History of fits, seizures, or loss of consciousness.
- Cognitive and learning: Challenges in learning new things, reading, writing, or doing simple calculations compared to peers.
- Speech and language: Difficulties in speaking or unusual speech patterns.
- Behavioural and social: Attention issues, interest in playing with other children

Self-care: Ability to feed themselves using hands or utensils.

The assessor is instructed to prioritize the mother's responses, but other family members' input is considered if needed.

### Ethics approval and consent to participate

Ethical approval for this study was obtained with yearly renewal from the following partner institutions: Indian Institute of Public Health-Bengaluru Institutional Ethics Committee (IIPHHB/TRCIEC/214/2021) on 20 May 2021; Sangath Institutional Review Board (GD_2022_77) on 4 April 2022; Ashoka University Institutional Ethics Committee (AUHREC/10062023/meeting2/002-7) on 10 June 2023; and St. John’s Research Institute Institutional Ethics Committee (IEC Study Ref No. 06/2024) on 8 February 2024. This study was reviewed and approved by the Technical Advisory Committee, Karnataka on 15 March 2022, and the received a letter of support from the National Health Mission, Haryana on 7 September 2022. We obtain written informed consent from all participants prior to data collection. Participation is voluntary, and participants are informed about the right to withdraw at any stage. They can choose to not answer specific questions. Privacy and confidentiality is maintained during the assessments, especially while requesting sensitive information, such as data related to intimate partner violence or harsh disciplining techniques. Only de-identified data is shared amongst partner institutions for analysis. The COINCIDE consortium encourages research groups interested in this area of work to contact the group for collaborations, access to study tools, and data. Ethical principles will be followed while sharing data with collaborators (e.g. only de-identified data will be shared after a formal memorandum of understandings is signed between parties to ensure that data is used for research purposes only and to maintain data privacy). 

### Dissemination of study findings

Study findings will be disseminated through peer-reviewed publications, conference presentations, invited talks, and through newsletters and social media pages of the COINCIDE team and its partners (For example: https://twitter.com/COINCIDE_2021 and the study website https://projectcoincide.org/ which is the main repository of all public information related to COINCIDE). Additionally, partner organisations have included information on the COINCIDE study on their institutional websites. A dissemination workshop will be organized towards the end of the study in 2026 to report key findings and the Theory of Change model to relevant stakeholders. 

## Discussion

Although the determinants of children’s cognitive and mental health outcomes are diverse and multifactorial, previous studies have largely focused on the effects of individual factors in isolation. This has led to biased estimates of the impact of critical exposures known to affect children’s outcomes, since they overlook the complex interplay of the multitude of co-occurring exposures that can modify each other’s effects. Furthermore, most studies focus on the early childhood period, even though risk factors for poor child outcomes continue to persist and accumulate into middle- and late-childhood, with potentially differential effects based on the child’s developmental stage ^105^. Specific pathways through which these diverse exposures interact with axes of health inequities, such as socioeconomic factors and gender, to result in inequities in child development outcomes, also remain unclear. These evidence gaps prevent us from designing effective, contextualized, and age-appropriate interventions to promote cognitive and mental health outcomes in children beyond the first three years of life.

The COINCIDE study, which brings together a multidisciplinary team of experts in areas of nutrition, environmental health, social science, child development, and advanced statistics, aims to overcome these limitations by evaluating the independent, interaction, and cumulative effects of nutritional status, psychosocial adversities, and environmental pollutant exposures on concurrent and prospective measures of cognitive development and mental health across the 3–10-year-age-range in urban and rural settings in India. By synchronising investments in two diverse urban and rural cohorts, the COINCIDE study will generate context-specific evidence necessary that could guide designing for future interventions. Evidence from the diverse cohorts in COINCIDE may highlight both universal *and* context-specific intervention targets specific to urban-rural and north-south regional differences in India, and relevant to the critical formative years of childhood that is least studied worldwide. Our findings may potentially be generalizable to other similar settings in the Global South.

### Strengths and limitations of the study

The strengths of the COINCIDE study lie in the rich historical dataset available for both the SPRING and MAASTHI birth cohorts from pregnancy until children are followed-up for this study. Therefore, this study will not only allow us to examine the concurrent and prospective effects of multiple exposures on children’s outcomes during the 3–10-year age range, but also investigate the long-term effects of perinatal and early childhood exposures. During the COINCIDE study period, the same tools and timelines will be used to collect data from both cohorts. This will allow us to characterize and directly compare the nature and effects of multiple exposures on child outcomes in diverse settings. The COINCIDE research infrastructure will therefore serve as a valuable resource for global health research, representative of understudied populations in the Global South during the critical middle- and late-childhood periods. It encompasses a wide range of assets, such as rich historical data from pregnancy to 10-years, stored biological samples, comprehensive cohort data from India, validated measures and standard operating protocols of nutritional, psychosocial, and environmental risk factor measurements, a well-trained team of field assessors, efficient data collection procedures, secure storage of exposure and outcome data, and linkages with the Health, Education, and Environmental Department data from the Government of India and personnel. Standard operating procedures are available in the local languages for all the COINCIDE study tools, which could be leveraged in other studies in similar settings.

Another strength of the COINCIDE study is its diverse sample from an LMIC setting representing different locations, cultures, behaviours, occupation, and diet (urban South and rural North India), and covers a broad age range, representing different developmental stages from pregnancy to late childhood. Cognitive development, one of the main outcome measures, is assessed using three different direct child measures (RCPM, DEEP, ASER) that examine several different domains of cognition. Additionally, the COINCIDE study has the potential to identify protective factors (nutritional status, parent-child interaction, early learning environment, etc.) that have previously been proposed to build resilience in the face of adversities ^106^. The rich COINCIDE dataset promises to be an invaluable resource for researchers and policymakers seeking to support children’s development in diverse settings of the Global South by building a nuanced and holistic understanding of the combined effects of multiple exposures on children’s outcomes through the first decade of life.

However, the following limitations should be noted when interpreting results. Historical cohort data that were collected before the start of the COINCIDE study is not uniform across the two sites. For example, although both cohorts collected data on maternal mental health, the SPRING cohort used the Patient Health Questionnaire-9, while the MAASTHI cohort used the Edinburgh Postnatal Depression Scale. In such cases, we will collect data on both the tools in both cohorts in a subset of the participants, and attempt to compare scores from the two tools using Spearman’s correlation. Preliminary analysis comparing EPDS and PHQ-9 scores noted high levels of correlation, thus indicating construct validity and consistency in maternal responses.

The second limitation emerges from the fact that data for most exposure and child mental health outcome measures are based on parent reports. Therefore, there will be an inherent bias that stems from indirect assessments and the recall of past events. Furthermore, while the comprehensive nature of exposure and outcome assessment is a strength, each household visit lasts for approximately two hours on average. This may lead to participant fatigue in some cases, potentially affecting engagement and data quality. Based on the results of our pilot study to evaluate the acceptability and feasibility of administering the COINCIDE study tools in participant households, we developed strategies to reduce assessment burden and fatigue by carefully selecting only those tools that are critical and essential for addressing the COINCIDE research questions, and providing breaks whenever necessary to reduce cognitive load in participants. Additionally, the more engaging and relatively shorter direct child assessments are completed faster (approximately in one hour, extending to 1.5 hours if the child requests for breaks), while questionnaires are directed only towards the adult caregiver. Finally, children are in different age brackets across the two cohorts, representing different developmental stages (3–8 years in MAASTHI, and 7–10 years in SPRING). While there is an overlap in certain historical measures (e.g., 24-hour diet recalls, developmental milestones tracking) which can be directly compared between sites, there are others that are missing in individual sites (e.g. maternal biological samples from the pregnancy period are only available in the MAASTHI cohort, while detailed assessments of early-life stress during infancy is only available in the SPRING cohort). This is expected for birth cohorts that were initially set up to answer different research questions. Therefore, in cases where relevant historical exposure or outcome measures are missing in particular cohorts, it will not be possible to directly compare results across sites. In these cases, we will conduct cohort-specific analysis. However, when possible, data from similar age groups across both cohorts will be pooled for analysis to increase statistical power and for direct comparison across sites.

## Conclusion

Despite the limitations highlighted above, the COINCIDE study is poised to make an important contribution towards an improved understanding of the individual and combined effects of a comprehensive set of exposures on child development and mental health across the first decade of life in an understudied population in the Global South, as well as demonstrating the pathways and their micro-drivers through which inequities in child outcomes emerge at the earliest stages of development using in-depth qualitative inquiry. Evidence from this study will be used to develop and refine multicomponent and contextualized interventions to promote children’s developmental potential across the early, middle, and late childhood periods. We anticipate that effective interventions that benefit the foundational years will continue to provide returns on investments throughout the life course ^107^, thereby helping improve health and economic outcomes and bolstering India’s demographic dividend.

## List of abbreviations

COINCIDE - Nutritional, Psychosocial, and Environmental determinants of Neurodevelopment and Child mental health; ECD - Early childhood development; NCF - Nurturing care framework; ACE - Adverse childhood experience; SES - Socio-economic status; MAASTHI – Maternal Antecedents of Adiposity and Studying the Transgenerational Role of Hyperglycemia and Insulin; SPRING – Sustainable Programme Incorporating Nutrition and Games; SDG - Sustainable development goals; LMIC - Low-middle income country; HIC – High-income country; NFHS - National Family Health Survey; IPT - Initial program theory ; ToC - Theory of Change; MUAC - Mid-upper arm circumference; IQ – Intelligent quotient; REDCap - Research Electronic Data Capture; DEEP- DEvelopmental Assessment on an E-Platform; RCPM - Raven’s Coloured Progressive Matrices; RSPM - Raven’s Standard Progressive Matrices; ASER - Annual State of Education Report; SDQ - Strengths and Difficulties Questionnaire; IAQ - Indoor Air Quality; PM - Particulate matter; CRP - C-Reactive Protein; AGP - Alpha(1)-acid glycoprotein (AGP); T3 – Triiodothyronine; T4 – Thyroxine; DAP - Dialkyl phosphate; FIES - Food Insecurity Experience Scale; EPDS - Edinburgh Postnatal Depression Scale; PHQ-9 - Patient Health Questionnaire; MSS - Maternal Social Support; CPRS - Child-Parent Relationship Scale; CPAS - Child Psychosocial Adversity Scale; CPAS IPV - Child Psychosocial Adversity Scale Intimate Partner Violence; FCI - Family care indicators; RBSK - Rashtriya Bal Swasthya Karyakram; DEIC - District Early Intervention Centre; IDI - In-depth interview; FGD - Focus group discussion; KII - Key informant interviews

## Declarations

**Consent for publication**

Not applicable.

## Ethics and consent

Ethical approval for this study was obtained with yearly renewal from the following partner institutions: Indian Institute of Public Health-Bengaluru Institutional Ethics Committee (IIPHHB/TRCIEC/214/2021) on 20 May 2021; Sangath Institutional Review Board (GD_2022_77) on 4 April 2022; Ashoka University Institutional Ethics Committee (AUHREC/10062023/meeting2/002-7) on 10 June 2023; and St. John’s Research Institute Institutional Ethics Committee (IEC Study Ref No. 06/2024) on 8 February 2024. This study was reviewed and approved by the Technical Advisory Committee, Karnataka on 15 March 2022, and the received a letter of support from the National Health Mission, Haryana on 7 September 2022. We obtain written informed consent from all participants prior to data collection.

## Data Availability

No data are associated with this article.
