# Supplementary material for: Protocol of the Nutritional, Psychosocial, and Environmental Determinants of Neurodevelopment and Child Mental Health (COINCIDE) study
Source: Wellcome Open Res. 2024 Nov 22;9:486. Originally published 2024 Aug 19. [Version 2] doi: 10.12688/wellcomeopenres.22817.2 (PMC11775445; doi:10.12688/wellcomeopenres.22817.2)
Supplement: Peer review history

# Reviewer report: Reviewer response for version 2

**DOI:** 10.21956/wellcomeopenres.25783.r112955

**Published:** 2025-01-28

**Authors:** Beena Koshy

**Affiliations:** 1 Christian Medical College, Vellore, India

All my concerns are addressed.

Is the study design appropriate for the research question?

Yes

Is the rationale for, and objectives of, the study clearly described?

Partly

Are sufficient details of the methods provided to allow replication by others?

Partly

Are the datasets clearly presented in a useable and accessible format?

Not applicable

Reviewer Expertise:

Child Development and Disability

I confirm that I have read this submission and believe that I have an appropriate level of expertise to confirm that it is of an acceptable scientific standard.

# Reviewer report: Reviewer response for version 1

**DOI:** 10.21956/wellcomeopenres.25123.r97652

**Published:** 2024-10-02

**Authors:** Esther Walton

**Affiliations:** 1 University of Bath, Bath, UK

This is an excellent protocol for the COINCIDE study, providing very detailed yet clear information on the aims, design, measures and key research questions of the study.

 Data collection across two regions with different demographic characteristics, with linkage to previous data collected in these samples, is a key strength of the proposed work. The selection criteria are clearly laid out. I also appreciate the collection of biological and both quantitative and qualitative data. Figure 2 describes the planned activities very clearly.

**I recommend clarifying the following:**

1. While it is mentioned that biological samples will be collected for genetic and epigenetic assessments, this is not included in Table 4. Is the plan to collect samples but not process them until later?
2. The Machine Learning section is arguable a bit brief. If space allowed, the authors could add more details, for example on how the training and testing samples will be split/defined.
3. I wondered whether more child self-reports could be included in this study. Most measures will be obtained using parental reports, but certain mental health traits (such as depression and other internalizing symptoms) can sometimes be better ascertained using self-reports.
4. I could not find who's funding this study (or previous waves of data collection).

In summary, this is a comprehensive description of a new ambitious study in India that aims to shed light into the nutritional, psychosocial, and environmental determinants of child mental health.

Is the study design appropriate for the research question?

Yes

Is the rationale for, and objectives of, the study clearly described?

Yes

Are sufficient details of the methods provided to allow replication by others?

Yes

Are the datasets clearly presented in a useable and accessible format?

Yes

Reviewer Expertise:

genetics, epigenetics, neuroimaging, biosocial pathways to child mental health, health trajectories across the lifespan

I confirm that I have read this submission and believe that I have an appropriate level of expertise to confirm that it is of an acceptable scientific standard.

# Response

**Published:** 2024-11-19

**Authors:** Debarati Mukherjee

**Affiliations:** Life course Epidemiology, Indian Institute of Public Health, Bengaluru, Public Health Foundation of India, Bengaluru, Karnataka, India

**Response to reviewer comments - Reviewer 2**

**This is an excellent protocol for the COINCIDE study, providing very detailed yet clear information on the aims, design, measures and key research questions of the study.** **Data collection across two regions with different demographic characteristics, with linkage to previous data collected in these samples, is a key strength of the proposed work. The selection criteria are clearly laid out. I also appreciate the collection of biological and both quantitative and qualitative data. Figure 2 describes the planned activities very clearly.**

**Response**: Thank you very much for your insightful and supportive review of our paper. We deeply appreciate your encouraging comments and are grateful for your constructive suggestions, which will enhance the clarity and impact of our work. We are truly grateful for your time and effort in reviewing our manuscript. We have provided a detailed point-wise response to each of your comments, including updated page numbers in the revised manuscript, where the changes can be viewed (V2).  

**I recommend clarifying the following:**

**While it is mentioned that biological samples will be collected for genetic and epigenetic assessments, this is not included in Table 4. Is the plan to collect samples but not process them until later?**

**Response:** Many thanks for this keen observation. As mentioned towards the end of our “Introduction” section (now under “Specific aims and hypotheses” section) we will be collecting biological samples for a range of analyses. Table 4 specifically includes the plans for the COINCIDE study, such as collection and processing of stored plasma and serum samples to measure biomarkers of micronutrients and heavy metals. The genetic, epigenetic, biochemical, and metabolomic assessments are part of our future plans. These analyses are beyond the scope of the current study, but collecting these samples will allow for their use in longitudinal analyses in future studies aimed at identifying novel biomarkers of child development during middle- and late-childhood, thereby maximizing the value of these data.  

**The Machine Learning section is arguable a bit brief. If space allowed, the authors could add more details, for example on how the training and testing samples will be split/defined.**

**Response: Thank you for your suggestion. We have now included the following in the main text as well:** *“We will develop a machine learning-based model to predict children’s cognitive, academic achievement, and mental health outcomes (scores based on direct child assessments and questionnaire-based tools) against predictor variables from the psychosocial, nutritional, and environmental domains. We will split the dataset for each study setting into training (70%) and validation (30%) datasets. Tree-based models are useful to model non-linearities and interactions between variables and would be our primary choice of algorithms. Random forests and gradient boosting models will be developed with internal cross-validation to tune the hyperparameters. Predictions from these algorithms on the validation dataset will be evaluated using measures of prediction accuracy such as R2 and root mean squared errors (RMSE).”* Please let us know if this level of detail is adequate for this protocol paper. We will be happy to furnish additional details if requested. The main manuscript which describes this data-driven approach will have a dedicated methods section which will provide a detailed description of the machine learning models and methods used to predict children’s outcomes.  

**I wondered whether more child self-reports could be included in this study. Most measures will be obtained using parental reports, but certain mental health traits (such as depression and other internalizing symptoms) can sometimes be better ascertained using self-reports.**

**Response:** We had the opportunity to pilot two child-reported questionnaires in older children (8-9-years-old; N = 32) in the SPRING cohort before the second sweep of data collection commenced. These were the Revised Child Anxiety and Depression Scale (RCADS) (47 items) and Revised Child and Youth Resilience Measurement (R-CYRM) (17 items). The parent-report versions of these questionnaires were simultaneously administered to children’s mothers by clinical psychologists. Based on the observations of the clinical psychologists and a senior research team member, the team took a decision **to not to use the child-reported questionnaires **for the following reasons:

1. *Challenges in children's comprehension of questionnaire items:* In the rural Rewari setting, children found it difficult to understand most of the questions. It was particularly challenging for them to understand the concepts of anxiety, obsessive compulsive disorder (OCD), and bad thoughts. Even with detailed explanations, they remained confused, and tended to select the same response option throughout the assessment.
2. *Assessment fatigue:* The lengthy questionnaire, especially the 47-item RCADS questionnaire, proved to be a significant assessment burden for children in this setting. This affected their responses, which could also have contributed to them consistently choosing the same response option, regardless of the question.
3. *Higher quality of parental (largely mothers) responses observed for the same questionnaires: *The supervision team agreed that parents found it easier to comprehend the questionnaire items when explanations were provided, resulting in more varied and potentially more appropriate responses. Since the parent-reported questionnaires were expected to yield higher quality data, the team made a decision to only proceed with the latter. Child reported questionnaires will be attempted in both the cohorts when children are older in future studies.

**I could not find who's funding this study (or previous waves of data collection).** **Response: **This work was supported by the DBT/Wellcome Trust India Alliance [IA/TSG/20/1/600023]. The urban MAASTHI birth cohort was established and followed up from 2016-2019 through funding by the DBT/Wellcome Trust India Alliance as an Intermediate fellowship awarded to Prof. Giridhara R. Babu. The rural SPRING cohort was funded from 2013-2018 by a Wellcome Trust Programme grant (0936115/Z/10/Z), followed by the Corporate Social Responsibility initiative of Madura Microfinance Ltd. from 2017- 2021 for a study aimed to develop the DEEP tool ^1-3^ ) through a study titled Integrated Assessment of Cognitive Development (REACH). **In summary, this is a comprehensive description of a new ambitious study in India that aims to shed light into the nutritional, psychosocial, and environmental determinants of child mental health.** **Response:** Thank you again for your detailed review. Your expertise has helped us refine our protocol paper.

**References:**

 1. Bhavnani S, Ranjan A, Mukherjee D, et al. A non-specialist worker delivered digital assessment of cognitive development (DEEP) in young children: a longitudinal validation study in rural India. *medRxiv* 2024: 2024.11. 04.24316724.

 2. Bhavnani S, Mukherjee D, Bhopal S, et al. The association of a novel digital tool for assessment of early childhood cognitive development, 'DEvelopmental assessment on an E-Platform (DEEP)', with growth in rural India: A proof of concept study. *EClinicalMedicine* 2021; **37**: 100964.

 3.  Mukherjee D, Bhavnani S, Swaminathan A, et al. Proof of Concept of a Gamified DEvelopmental Assessment on an E-Platform (DEEP) Tool to Measure Cognitive Development in Rural Indian Preschool Children. *Front Psychol* 2020; **11**: 1202.

# Reviewer report: Reviewer response for version 1

**DOI:** 10.21956/wellcomeopenres.25123.r96519

**Published:** 2024-09-19

**Authors:** Beena Koshy

**Affiliations:** 1 Christian Medical College, Vellore, India

It is important to identify factors that can help to optimize mid-late childhood, thus this study is relevant.

 Some concerns

1. Abstract: Early childhood word is used. Here the age group is 3-9 years. So, this use needs to be justified
2. It is good to clearly state objectives and hypotheses for this study at the end of Introduction.
3. SPRING cohort is an interventional cohort – how can this be utilized for baseline analysis and compared with other baseline cohorts?
4. Table 2 – Under dietary diversity, how can venous blood be used to measure dietary diversity?
5. Table 2 – Is outcome measurement on BSID-III or BSID -II? Both have been mentioned – better to use one version for comparison purposes.
6. Table 2 – Difference in units (d) – is this difference in SD or composite score difference
7. If composite score difference, justify use of 1.14 units difference in dietary diversity assessment and 1.15 units in ambient air pollution– clinically and developmentally this is not significant
8. How will be socio-economic status measured and what questionnaire will be used? Were similar questionnaires or measures used in both cohorts in the past?
9. Good to give description of RBSK checklist in the manuscript
10. Are there norms for DEEP in this age group for Indian population – how do you plan to interpret this outcome/measure?
11. Let me suggest that ASER is not a cognitive assessment but more of an academic achievement tool.
12. Rather than a specific age follow-up, the study proposes cross-sectional analyses at two points for both cohorts – what is the justification for the second sweep analysis as it happens just a year after the first analysis?
13. It is good that criteria for referral is clearly indicated for maternal and childhood measures.

Is the study design appropriate for the research question?

Yes

Is the rationale for, and objectives of, the study clearly described?

Partly

Are sufficient details of the methods provided to allow replication by others?

Partly

Are the datasets clearly presented in a useable and accessible format?

Not applicable

Reviewer Expertise:

Child Development and Disability

I confirm that I have read this submission and believe that I have an appropriate level of expertise to confirm that it is of an acceptable scientific standard, however I have significant reservations, as outlined above.

# Response

**Published:** 2024-11-19

**Authors:** Debarati Mukherjee

**Affiliations:** Life course Epidemiology, Indian Institute of Public Health, Bengaluru, Public Health Foundation of India, Bengaluru, Karnataka, India

**Response to reviewer comments - Reviewer 1** **It is important to identify factors that can help to optimize mid-late childhood, thus this study is relevant.** **Response: **We thank the reviewer for their time and effort in reviewing our manuscript. We highly value their meticulous attention to detail and insightful comments, which we believe have significantly enhanced the quality of our manuscript. We have provided a detailed point-wise response to each comment, and made changes in the revised manuscript (Manuscript v2).

**Some concerns**

**Abstract:** **Early childhood word is used. Here the age group is 3-9 years. So, this use needs to be justified**

**Response: **Thank you for raising this important point. We agree that the term "middle- and late-childhood" more accurately represents the study's focus on the 3–10-year age group. We have now revised the sentence to replace *early *with *middle- and late-*childhood. We have also revised this throughout the manuscript. **Please see the following edit in the “Abstract”:** *“Thematic analysis of in-depth interviews and focus group discussions will explore pathways to middle-and late-childhood development inequities.”*  

**It is good to clearly state objectives and hypotheses for this study at the end of Introduction.**

**Response:  **Thank you for this suggestion. In the “Introduction”, we have now added a new subsection titled “Specific aims and hypotheses” to address this, as detailed below.

***Hypothesis*** *We hypothesize that *

- ​​​​​​​​​​​​​​ *Specific adversities experienced within the nutritional, psychosocial, and environmental domains will have differential impacts on children’s cognitive and/or mental health outcomes. *
- *Each type of adversity will have a ‘sensitive window of exposure’ wherein exposure to this factor in this critical time window will have long-lasting effects on child outcomes during middle- and late-childhood.*
- *Increasing number of adversities will be associated with poor cognitive and mental health outcomes across all age groups in both urban and rural settings. However, the nature of adversities will differ across the urban and rural settings*
- *The psychosocial environment of the child will moderate the effects of adverse nutritional and environmental pollutant exposures during middle- and late-childhood. Specifically, a nurturing and responsive environment will protect against the harmful effects of adverse nutritional or environmental pollutant exposures, while an abusive or neglectful environment will exacerbate their negative effects.*
*In addition to the hypothesis-driven research described above, we will also attempt to identify the combination of the most important predictors of child outcomes in different age groups and settings using a data-driven machine learning approach. The outcome will be tested in a naïve test sample that does not contribute to training the machine learning models.*  
**SPRING cohort is an interventional cohort – how can this be utilized for baseline analysis and compared with other baseline cohorts?**
**Response: **Thank you for raising this critical point as we had anticipated this concern during the planning phase. Indeed, the SPRING cohort was originally designed as an interventional cohort, intended to assess the effects of nutrition, parenting, and play interventions on child health and development outcomes. However, analyses from the SPRING study revealed that the interventions had no significant effect on children’s outcomes. ^1^ As a result, the SPRING cohort has been treated as an observational cohort in subsequent studies with robust baseline data, allowing for comparisons with other observational cohorts.  
**Table 2 – Under dietary diversity, how can venous blood be used to measure dietary diversity?**
**Response: **Thank you for flagging this error. The COINCIDE study will use the household level dietary diversity questionnaire developed by the Food and Agriculture Organization (FAO) to assess dietary diversity. ^2^ However, this factor is no longer included in Table 2, as we have focused on using the Raven’s Coloured Progressive Matrices as the outcome measure of interest, instead of the Bayley’s Scales of Infant Development-III scores, to calculate sample sizes. This was done in response to your next query (number 5), and to keep our study aligned with a child outcome measure which was more appropriate for middle- and late-childhood. We could not identify any previous studies that used dietary diversity as an exposure measure linked to RCPM as the outcome measure, to facilitate sample size calculation.  
**Table 2 – Is outcome measurement on BSID-III or BSID -II? Both have been mentioned – better to use one version for comparison purposes.** **Response: **In the first draft of the protocol paper, we had used the BSID-III or BSID-II as an overall measure of child development as the outcome, depending on the availability of published literature on the relevant exposure-outcome relationship. However, we recognized that BSID is a measure of early child development, and therefore not directly relevant to the COINCIDE study. Therefore, in the updated current version, we recalculated the sample sizes using the Raven’s Coloured Progressive Matrices (RCPM) score as the outcome measure, which is also the primary measure of cognitive development in the COINCIDE study in 4-10-year-old children. The updated sample sizes based on RCPM as the outcome measure is presented in Table 2 for the nutritional and psychosocial domains.    
**Table 2 – Difference in units (d) – is this difference in SD or composite score difference**
**Response: **Thank you for pointing this omission in our description of Table 2. The difference in units (d) refers to the difference in Standard Deviations (SD), which holds more clinical relevance. This has now been clarified in the footnote for Table 2.  
**If composite score difference, justify use of 1.14 units difference in dietary diversity assessment and 1.15 units in ambient air pollution– clinically and developmentally this is not significant**
**Response: **As described in the response to Q6, the difference is in Standard Deviation (SD) units and not in composite scores.  
**How will be socio-economic status measured and what questionnaire will be used? Were similar questionnaires or measures used in both cohorts in the past?**
**Response: **Socio-economic status (SES) will be calculated using Principal Component Analysis score derived using the following variables: maternal and paternal education and occupation, crowding (number of household members divided by the number of rooms used for sleeping), source of drinking water (piped into household vs other), material of the walls, floor, and roof, toilet facility (owned versus shared), household asset list (Electricity, Mattress, Pressure cooker, Chair, Cot or Bed, Table, Electrical fan, Radio or Transistor, Black & White Television, Colour Television, Sewing Machine, Mobile Telephone, Landline Telephone, Internet, Computer or tablet, Refrigerator, Air conditioner / Cooler, Washing Machine, Watch or Clock, Bicycle, Motorcycle or Scooter, Auto or Tempo, Car, Water-pump, Thresher, Tractor), ownership by any household member of real estate, bank account, or a government-authorized card identifying the family as living below the poverty line, and presence of a domestic servant in the house. The same questionnaire has been used in both the cohorts to estimate household-level SES in the COINCIDE study. Historically, data collection methods were not harmonized in the MAASTHI and SPRING cohorts before the COINCIDE study. Previously, the SPRING cohort collected SES data using a modified version of the Demographic and Health Survey tool, followed by Principal Component Analysis (PCA) of data on parental and household demographics, and animal & other asset ownership. ^3^ In contrast, the MAASTHI cohort collected SES data using the Kuppuswamy Scale. ^4^ Given these differences in data collection methods before the commencement of the COINCIDE study, as well as the differences in children’s age profile across the two cohorts, the team has decided to conduct all analysis independently for each cohort, and compare and contrast the summary results – not pool data from both cohorts into a single analytic dataset unless harmonized data is available for the same child age across both the cohorts.  
**Good to give description of RBSK checklist in the manuscript**
**Response: **Thank you for your suggestion. We have now included a sub-section and made the following edits: *“ **Developmental milestones/screening*** *Although not an outcome of our study, we included a questionnaire to screen for developmental delays and disabilities to identify children who would benefit from a referral for further assessment, diagnosis, and treatment as required. We used the Rashtriya Bal Swasthya Karyakram (RBSK) questionnaire for this purpose. This is a comprehensive screening tool aimed at early detection of the 4 'D's: defects at birth, diseases, deficiencies, and developmental delays, including disabilities, in children aged 0-18 years, to facilitate timely intervention and treatment.* *The RBSK checklist is a structured tool used to screen children, and includes a series of questions designed to assess various domains of child health:*
- *Vision and auditory: Difficulty in seeing at day/ night; difficulty in hearing (without hearing aid)*
- *Motor development: Delays in walking, stiffness or weakness in limbs.*
- *Neurological concerns: History of fits, seizures, or loss of consciousness.*
- *Cognitive and learning: Challenges in learning new things, reading, writing, or doing simple calculations compared to peers.*
- *Speech and language: Difficulties in speaking or unusual speech patterns.*
- *Behavioural and social: Attention issues, interest in playing with other children*
- *Self-care: Ability to feed themselves using hands or utensils.*

*The assessor is instructed to prioritize the mother's responses, but other family members' input is considered if needed.”*  

**Are there norms for DEEP in this age group for Indian population – how do you plan to interpret this outcome/measure?**

**Response: **Thank you for this question. The DEEP tool has now been used on ~1080 children in urban and ~1200 children in rural India through other studies, and norms are being developed using these data. Once available, data from the COINCIDE study will be compared with these norms to identify poor performers in the cognitive domain. We also want to mention that COINCIDE study partners (Sangath India) have published a preprint that describes methodology that will be used to derive a DEEP score, which in turn will be used on data from 2000 children in Delhi to develop the India norms. ^5^  

**Let me suggest that ASER is not a cognitive assessment but more of an academic achievement tool.** **Response: **Thank you for sharing this insight. We agree with your suggestion. Therefore, we have indicated DEEP and RCPM as measures of cognitive development in the updated version of the manuscript, and ASER as a measure of academic achievement. However, since the latter also involve aspects of cognitive functioning, such as skills related to thinking, learning, problem-solving, memory, etc., we had initially broadly classified ASER as a tool to measure cognitive development.  

**Rather than a specific age follow-up, the study proposes cross-sectional analyses at two points for both cohorts – what is the justification for the second sweep analysis as it happens just a year after the first analysis?**

**Response: **The second sweep in COINCIDE has been timed to occur 15-18 months after the first sweep, which we believe is a significant time lag, even more so for the young 3-6-year age group in the MAASTHI cohort. This interval is crucial for understanding the dynamic cognitive, social, and emotional development that occurs rapidly during middle- and late-childhood. Capturing these developmental shifts during this critical period will provide nuanced insights into growth and development trends during these critical periods, as well as the relevant exposures that influence these outcomes. Further, the analysis plan also includes prospective cohort analysis of child outcomes using historical data available in both the cohorts since pregnancy.  To share an example, we have collected data on depressive symptoms in mothers since pregnancy in the MAASTHI cohort. This historical data, along with the cross-sectional data on depressive symptoms collected in the COINCIDE study, will be used to study the role of maternal depressive symptoms since pregnancy on child mental health outcomes when children are 4-8-years-old. Similarly, we have collected data on the home learning environment since children were 1-year-old in the SPRING cohort. This prospective data will be analysed to understand both how this exposure changes as children grow up in rural India, and the long-term role of the home environment on children’s cognitive and mental health outcomes during the late-childhood period. We also referred to several well-established cohorts including the **DRAKENSTEIN Child Health Study** in South Africa that followed children from birth with multiple assessments through infancy and early childhood (36, 42, 54, 60, 66 months). ^6^ Further, the **Growing Up in Australia: The Longitudinal Study of Australian Children (LSAC)** followed children from infancy with assessments every 2-3 years. ^7^ The **MAL-ED (Malnutrition and Enteric Disease) cohort** study in India ^8^ followed up children at 3, 5, 7 and 9 years of age. **The Avon Longitudinal Study of Parents and Children (ALSPAC) **collected data frequently from birth through adolescence, including key developmental assessments at ages 7, 8, and older. ^9,10^ The **UK Millennium Cohort Study (MCS) **also included follow-ups at regular intervals from early childhood through adolescence, such as at ages 3, 5, 7, and 11. ^7^ The COINCIDE effort aims to generate high-quality data and insights on child development and its determinants across the first decade of life in India.        

**It is good that criteria for referral is clearly indicated for maternal and childhood measures.**

**Response: **Thank you for commending our referral criteria for maternal and childhood measures; we appreciate your attention to this important detail.  

**References:**

 1.  Kirkwood BR, Sikander S, Roy R, et al. Effect of the SPRING home visits intervention on early child development and growth in rural India and Pakistan: parallel cluster randomised controlled trials. *Front Nutr* 2023; **10**: 1155763.

 2. Kennedy G, Ballard T, Dop M. Guidelines for measuring household and individual dietary diversity. 2011.

 3.Bhopal S, Roy R, Verma D, et al. Impact of adversity on early childhood growth & development in rural India: Findings from the early life stress sub-study of the SPRING cluster randomised controlled trial (SPRING-ELS). *PLoS One* 2019; **14**(1): e0209122.

 4. Singh T, Sharma S, Nagesh S. Socio-economic status scales updated for 2017. *Int J Res Med Sci* 2017; **5**(7): 3264-7.

 5. Bhavnani S, Ranjan A, Mukherjee D, et al. A non-specialist worker delivered digital assessment of cognitive development (DEEP) in young children: a longitudinal validation study in rural India. *medRxiv* 2024: 2024.11. 04.24316724.

 6. Donald KA, Hoogenhout M, du Plooy CP, et al. Drakenstein Child Health Study (DCHS): investigating determinants of early child development and cognition. *BMJ Paediatrics Open* 2018; **2**(1): e000282.

 7. Gray M, Smart D. Growing Up in Australia: The Longitudinal Study of Australian Children: A Valuable New Data Source for Economists. *Australian Economic Review* 2009; **42**(3): 367-76. 8. Koshy B, Srinivasan M, Gopalakrishnan S, et al. Are early childhood stunting and catch-up growth associated with school age cognition?—Evidence from an Indian birth cohort. *PLOS ONE* 2022; **17**(3): e0264010.

 9. Boyd A, Golding J, Macleod J, et al. Cohort Profile: the 'children of the 90s'--the index offspring of the Avon Longitudinal Study of Parents and Children. *Int J Epidemiol* 2013; **42**(1): 111-27.

 10.Golding J, Pembrey M, Jones R. ALSPAC--the Avon Longitudinal Study of Parents and Children. I. Study methodology. *Paediatr Perinat Epidemiol* 2001; **15**(1): 74-87.
